# Predicting Malignant Transformation in Oral Leukoplakia: A Multilayer Perceptron Approach Incorporating Clinicopathological Features and DNA Content

**DOI:** 10.1111/jop.70084

**Published:** 2025-11-11

**Authors:** Guilherme Iani Pontes, Anna Luíza Damaceno Araújo, Andresa Borges Soares, Saman Warnakulasuriya, André Luis Santana de Freitas, Caroline Gennari Stevão, Marcelo Sperandio, Matheus Cardoso Moraes

**Affiliations:** ^1^ Institute of Science and Technology Federal University of São Paulo (ICT‐UNIFESP) São José dos Campos São Paulo Brazil; ^2^ Head and Neck Surgery Department University of São Paulo Medical School São Paulo São Paulo Brazil; ^3^ Department of Surgery, Stomatology, Pathology, and Radiology, Area of Pathology, Bauru School of Dentistry University of São Paulo (FOB USP) Bauru São Paulo Brazil; ^4^ Faculty of Dentistry, Oral and Craniofacial Sciences King's College London London UK; ^5^ WHO Collaborating Centre for Oral Cancer London UK; ^6^ Faculdade São Leopoldo Mandic Campinas São Paulo Brazil

**Keywords:** DNA content, DNA content analysis, epithelial dysplasia, machine learning, malignant transformation, OPMD, oral cancer, oral leukoplakia, ploidy, risk prediction models

## Abstract

**Background:**

Oral leukoplakia (OL) is a potentially malignant disorder of the oral mucosa. Accurate prediction of malignant transformation (MT) remains a clinical challenge. This study aimed to develop and evaluate a machine learning model that integrates histopathological, demographic, and DNA content features to predict MT risk in OL.

**Methods:**

We conducted a retrospective cohort study of 97 OL cases—18 with confirmed MT and 79 non‐transformed controls—selected from a larger series. Each case included clinicopathological features, and DNA content data obtained by flow cytometry for cell cycle phases (G1, S‐phase, G2 and excess DNA beyond the tetraploid region [4cER]). All cases had a minimum 5‐year follow‐up or histologically confirmed transformation. A multilayer perceptron (MLP) model was trained on 27 features. Stratified five‐fold cross‐validation and minority class oversampling (positive filling) were used to improve learning and mitigate data imbalance. Performance was evaluated using accuracy, sensitivity, specificity, F1‐score, AUC, and Kaplan–Meier survival analysis.

**Results:**

Significant predictors of MT included 4cER (*p* = 0.005), G2 phase (*p* = 0.04), dysplasia grading (*p* = 0.003), and inflammatory infiltrate (*p* = 0.01). The optimized model yielded 72% sensitivity, 96% specificity, and an AUC of 85.4%. Survival analysis showed significantly poorer outcomes in the high‐risk cases predicted by the model (*p* < 0.0001).

**Conclusion:**

Integrating DNA content analysis with machine learning provides an objective and clinically useful model to stratify malignant risk in OL, complementing conventional histopathology and supporting personalized patient management.

## 1 Introduction

Assessing the risk of malignant transformation (MT) in oral potentially malignant disorders (OPMDs) remains a central challenge in oral medicine practice. Despite the routine use of epithelial dysplasia grading systems, their predictive reliability is limited by interobserver variability, modest reproducibility, and diagnostic subjectivity [1, 2]. This is particularly concerning given the substantial risk of transformation associated with oral leukoplakia (OL), the most common OPMD. A recent meta‐analysis estimated a pooled transformation rate of approximately 7.9%, with higher rates observed in high‐grade lesions and specific anatomical sites [3].

The 2021 World Health Organization Collaborating Centre for Oral Cancer Consensus Report redefined the classification and diagnostic criteria for OPMDs, emphasizing the need for more objective and quantifiable markers to inform clinical management [4]. In this context, machine learning (ML) has emerged as a powerful tool to enhance diagnostic accuracy across various domains of pathology, including oral mucosal disease [5, 6, 7].

Among the candidate biomarkers, DNA content analysis has shown promise in identifying aneuploidy—an early hallmark of carcinogenesis—particularly when assessed by flow cytometry [8, 9, 10]. A previous study demonstrated that flow cytometric ploidy analysis, when combined with ML, could improve sensitivity in detecting high‐risk OL cases [6]. Parallel investigations into specific dysplastic features have also identified key predictors of transformation, including loss of epithelial cohesion, prominent nucleoli, and inflammatory infiltrate [11].

Building on these insights, the present study aimed to develop and validate a supervised ML model, specifically a multilayer perceptron (MLP), for predicting MT in OL. The model integrates clinicopathological features, and DNA content parameters to generate a risk stratification framework. By leveraging long‐term follow‐up data and robust cross‐validation techniques, this work seeks to improve the accuracy, consistency, and clinical utility of MT risk prediction in OPMD management.

## 2 Methods

This study was approved by the research ethics committee of the Faculdade São Leopoldo Mandic (#2.380.013, 13/11/2017) and the State University of Campinas (UNICAMP #3.800.169, 14/01/2020).

The REMARK (reporting recommendations for tumor marker prognostic studies) guidelines were used to design this report, which falls within the definition of a phase 3 early cancer detection biomarker investigation [12].

The machine learning model was developed according to the TRIPOD + AI (Transparent Reporting of a multivariable prediction model for Individual Prognosis or Diagnosis using Artificial Intelligence) statement [13].

### 2.1 Study Design and Primary Outcomes

This was a retrospective cohort study designed to develop and validate a clinical prediction model for MT in OL. The primary outcomes were to assess model performance metrics—sensitivity, specificity, precision, F1‐score, and area under the receiver operating characteristic curve (AUC)—measured via stratified five‐fold cross‐validation. Additionally, Kaplan–Meier survival analysis was used to assess differences in transformation‐free survival between model‐predicted high‐ and low‐risk groups.

#### 2.1.1 Sample Sourcing

We identified 878 formalin‐fixed, paraffin‐embedded (FFPE) oral mucosal incisional biopsies diagnosed with OL in the pathology archives of Faculdade São Leopoldo Mandic spanning from 2005 to 2018. Among these patients, 35 were found to have undergone MT, as previously documented [9]. The selection process involved reviewing clinical and pathology diagnoses for terms such as keratosis, hyperkeratosis, leukoplakia, white patch, white plaque, white lesion, dysplasia, and atypia. These inclusion criteria supported the diagnosis of leukoplakia based on clinical and histopathological features described in pathology reports and clinical request forms retrospectively, aligning with current consensus classification [4]. Exclusion criteria were then applied, as follows:

Exclusion criteria comprised confounding lesions like candidiasis, frictional keratosis (whenever history of trauma was stated in the clinical request form), lichenoid reaction, lichen planus, or any other OPMD to reduce or eliminate the risk of misdiagnosing OL. Additionally, patients with lesions on the lip (e.g., actinic keratosis) or a prior diagnosis of oral squamous cell carcinoma (OSCC) were excluded. Paraffin blocks from each case were inspected for available tissue, with those missing or lacking sufficient tissue for analysis being excluded. In cases of multiple biopsies from the same individual, the first chronologically occurring lesion with the highest risk (based on DNA content) was selected using a “first worst” approach [8].

Based on the identification of 878 cases, 35 underwent MT, resulting in an overall MT rate of 4% [9]. Among the cases that transformed, 20 had sufficient material for DNA content analysis, while 2 failed to yield interpretable histograms (as defined in Dominguete et al. [6]), resulting in a final sample of 18 cases.

For the cases that did not progress to cancer, 120 were randomly selected for DNA content analysis (Microsoft Excel RAND function). However, 41 were excluded; 22 had insufficient tissue on the paraffin block, and 19 had material that failed to yield interpretable histograms. The final sample of cases that did not undergo MT was 79.

#### 2.1.2 Follow‐Up Data

Follow‐up to identify malignant lesions involved reviewing all cases diagnosed as OSCC from 2005 to 2022 and comparing them with previously identified cases of leukoplakia using criteria such as hospital number, name, date of birth, sex, and lesion site. Matched cases underwent a thorough review of histopathological reports and biopsy request forms to confirm patient identity using demographic data such as biopsy material origin (city, state, surgeon, etc.) and patient address.

Additional follow‐up data was obtained from the Hospital‐based Cancer Registry of the Hospital das Clínicas of the State University of Campinas (HC‐UNICAMP). Patients diagnosed with leukoplakia in 2016, 2017, and 2018 are currently under follow‐up by the oral medicine service of Faculdade São Leopoldo Mandic, with a minimum follow‐up period of 5 years.

#### 2.1.3 Nuclei Suspensions

The description of nuclei suspensions preparation is detailed elsewhere [6, 14]. Briefly, the biopsy specimens underwent tissue analysis of the entire epithelium, grossly microdissected and then sliced into multiple 50 μm sections. The sections were deparaffinized, rehydrated, and incubated with protease to release nuclei [14]. After filtration and centrifugation, the nuclei were resuspended in PBS. The nuclear density was adjusted to 10^5^–10^6^ nuclei per 500 μL and left overnight at 4°C. The nuclear suspensions were then gently agitated and stained with propidium iodide (PI) for flow cytometry analysis using a Gallios cytometer. Fluorescence was detected and quantified using a 661–683 nm fluorescence detector (FL4 filter), with debris gated out before analysis. Criteria for analysis followed recommendations by Ormerod et al. [15], excluding criteria specific to neoplastic tissue. Each preparation batch included positive and negative control samples for quality assurance.

A minimum of 2000 epithelial nuclei were assessed and samples with a diploid peak (G1) coefficient of variation (CV) greater than 7% were excluded from the analysis, unless grossly multiploid and with a high 4c exceeding rate (4cER)– a measure of abnormal DNA content above tetraploid levels.

Histogram data handling was performed as described by Dominguete et al. [6]. Briefly, peaks of nuclei at the G1, S‐phase, G2 and exceeding G2 regions (4cER) were manually established based on the symmetry of half‐peaks and the equivalence of peak width between G1 and G2. S‐phase and 4cER were defined by exclusion, following the establishment of G1 and G2 gates.

#### 2.1.4 Identification of Individual Features of Dysplasia in the Sample

Two calibrated examiners (MS, and ABS) [11] reviewed all the pathology sections by consensus, based on the 4th Edition of the WHO/IARC classification of Head and Neck Tumours [16], which included architectural features, namely (1) irregular epithelial stratification, (2) loss of polarity of basal cells, (3) drop‐shaped rete ridges, (4) increased number of mitotic figures, (5) abnormally superficial mitotic figures, (6) premature keratinization in single cells, (7) keratin pearls within rete ridges, (8) loss of epithelial cell cohesion and (9) cytologic features, namely (1) abnormal variation in nuclear size, (2) abnormal variation in nuclear shape, (3) abnormal variation in cell size, (4) abnormal variation in cell shape, (5) increased nuclear/cytoplasmic ratio, (6) atypical mitotic figures, (7) increased number and size of nucleoli, (8) hyperchromasia. To these parenchymal criteria, a stromal component was added, namely chronic inflammatory infiltrate, as present (1) or absent (0).

All features (columns) were annotated on a spreadsheet as being absent (0) or present (1) for each patient (rows). The outcome (MT) was included on the spreadsheet as not transformed (0) or with MT (1).

### 2.2 Data Analysis

#### 2.2.1 Correlation Analysis

Spearman correlation was used to ascertain the unadjusted association between each candidate predictor (attribute) and the outcome (MT). Only the correlations with *p* < 0.05 were considered significant and the strength of the correlations was interpreted as proposed by Landis and Koch [15] as 0–0.2 (slight), 0.2–0.4 (fair), 0.4–0.6 (substantial), 0.6–0.8 (strong) and 0.8–1 (near perfect).

#### 2.2.2 Multivariate Analysis

Kernel Density Estimation (KDE) plots were used to visualize the relationship between each feature and the distribution of the two subpopulations (MT and no MT). The Gaussian kernel was used due to its smoothness properties and its ability to capture the underlying distribution of the data. Estimation Procedure: for each subpopulation (high‐risk and low‐risk), the probability density function (PDF) was estimated using the KDE method by placing a kernel at each data point and summing the contributions from all kernels to obtain the overall PDF estimate for that subpopulation. The estimated PDFs were normalized for both subpopulations so that the integral over the entire domain was equal to one, ensuring they represented valid PDFs. The comparison between the high‐risk and low‐risk subpopulations’ distributions for each candidate predictor (attribute) was assessed visually through density plots.

#### 2.2.3 Multilayer Perceptron (MLP) Model to Classify High‐Risk and Low‐Risk Lesions

Data Preprocessing. Missing values were imputed or samples with missing values were excluded. Categorical variables were encoded, and numerical variables were standardized or normalized.

### 2.3 Model Development

The model was developed according to the TRIPOD + AI statement [13] and current guidelines for artificial intelligence in healthcare applications [17, 18].

Data for this study was extracted from the selected OL cases and the dataset consisted of 97 samples, each characterized by 27 attributes, which included age, sex, intraoral site, individual OED features, and DNA content values.

#### 2.3.1 Data Preprocessing

Before training, data preprocessing was performed to ensure information quality. Features with significant missing data that could not be imputed were excluded.

#### 2.3.2 Model Architecture and Parameters

An MLP neural network was used for classification. The network architecture included an input layer with 27 neurons, an output layer with 2 neurons, and three hidden layers with 20, 12, and 12 neurons, respectively. Categorical cross‐entropy was used to compute the loss function, and the Adam optimizer was employed with a learning rate of 0.005. The batch size was set at 25% of the data, and training occurred over 1000 epochs. The above parameter values were fine‐tuned during implementation, focusing on the best performance.

#### 2.3.3 Training Strategies and Evaluation Process

**FIGURE 1:**
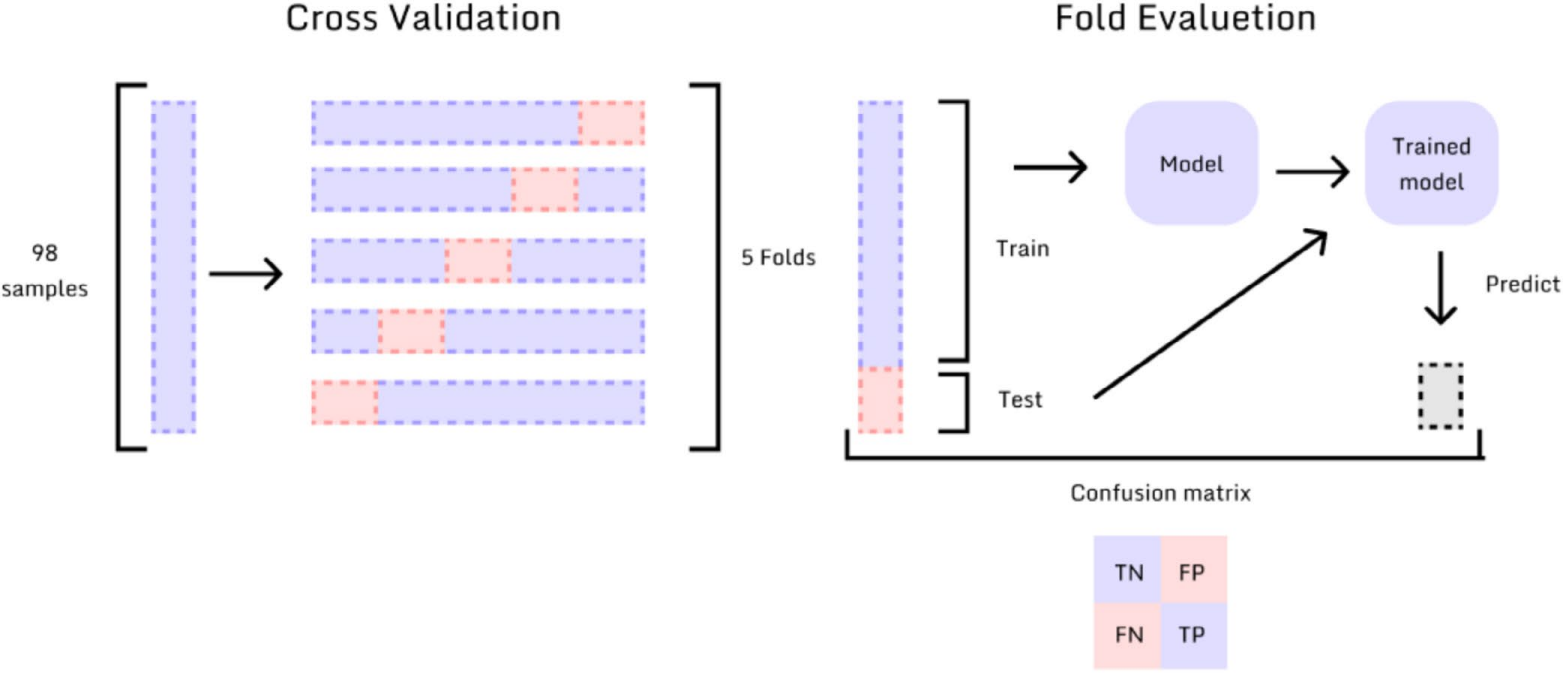
Illustration of a 5‐fold cross‐validation method, in which dataset and corresponding classes are proportionally divided to train and evaluate the model in five different dataset distribution contexts.

**FIGURE 2:**
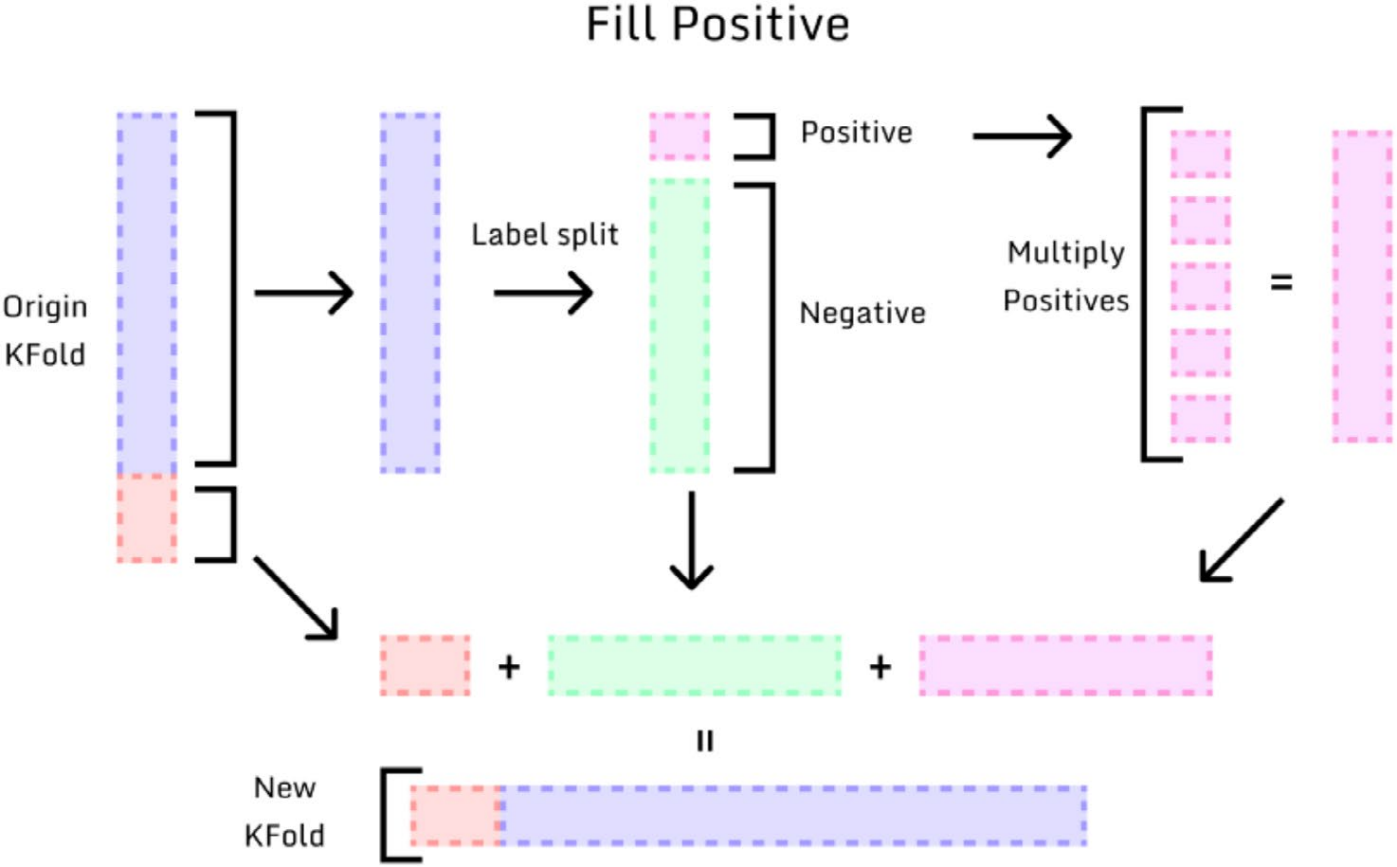
Illustration of the StratifiedKFold with positive filling strategy; in this strategy the small positive samples are multiplied to balance the dataset classes, hence, improving the model's learning classes by dedicating even energy for both classes' learning process.

Model evaluation was conducted using cross‐validation, repeating training and testing five times with alternating datasets (Figure 1). Three different training strategies were applied: (1) Simple k‐fold cross‐validation, in which sample classes are proportionally divided considering their original size in each fold; (2) Stratified Fold, which preserves label proportions across folds for imbalanced data; (3) StratifiedKFold with positive filling. In this strategy, the positive filling did not involve synthetic generation of artificial samples. Instead, it replicated the minority class instances (i.e., the 18 MT cases) during training so that each batch contained a balanced number of cases from both classes. This ensured that the model allocated equal attention to both classes during training epochs. This strategy was applied exclusively within the training folds during cross‐validation, ensuring equal exposure to both classes (Figure 2).

### 2.4 Model Performance

The performance of the model was measured based on the average performance metrics across all folds. The performance metrics included accuracy, sensitivity (recall), specificity, precision, F1‐Score and area under the curve (AUC). These were calculated using the corresponding test samples for each cross‐validation iteration and averaged across methods. In each iteration, model predictions were saved and aggregated to build Kaplan–Meier curves, allowing a detailed analysis of survival over time for different predicted groups.

### 2.5 Risk Assessment

Survival analysis. Kaplan–Meier survival analysis and Mantel‐Cox log‐rank test were used to compare DNA content‐based risk groups regarding time to MT (*p* < 0.05). Cases were censored on the date when an oral carcinoma was diagnosed or at the end of the follow‐up period (31/12/2023).

From the Kaplan–Meier curves, positive and negative predictive values for MT of DNA content‐based risk were calculated as well as sensitivity, specificity, odds ratio and 95% confidence intervals.

The statistical calculations were performed on GraphPad Prism (version 10) considering *p* < 0.05 as significant.

## 3 Results

### 3.1 Clinical and/or Demographic Features

The mean age of the patients who underwent MT was 57.9 years (SD = 13.4), with a mean time to MT of 13.6 months (SD = 17.8). Table 1 lists the clinicopathological data of the analysed cases. No significant correlation was found between age groups and MT (*p* > 0.08), nor was there a correlation between gender and transformation (*p* = 0.39).

**TABLE 1:** Clinicopathological, DNA content, and model‐based features stratified by transformation status. Includes frequency of dysplasia features, mean cell fractions at each cell cycle phase, and predictive performance of the main MLP model.

Variable	Category	Transformed (*n* = 18)	Non‐transformed (*n* = 79)	Statistics
Gender		Male: 5 (28%) Female: 9 (50%) NK: 4 (22%)	Male: 39 (49.4%) Female: 40 (50.6%)	Descriptive
Age‐group (years)	< 40	0	11 (14%)	Descriptive
	40–49	3 (16%)	8 (10%)	
	50–59	8 (45%)	20 (25%)	
	60–69	3 (17%)	25 (32%)	
	> 70	4 (22%)	15 (19%)	
Anatomical site	Buccal mucosa	0	10 (13%)	Descriptive
	Gingiva/alveolar ridge	2 (11%)	17 (21%)	
	Palate	3 (17%)	6 (7.6%)	
	Floor of mouth	1 (5.5%)	13 (16.4%)	
	Retromolar	1 (5.5%)	5 (6.4%)	
	Tongue	7 (39%)	22 (28%)	
	NK	4 (22%)	6 (7.6%)	
Histology	Most prevalent dysplasia features	**Increased number and size of nucleoli (72%)** Loss of polarity (67%) **Nuclear size variation (61%)** **Hyperchromasia (56%)**	Loss of polarity (53%) **Drop‐shaped rete ridges (52%)** **Irregular stratification (51%)** Increased nuclear/cytoplasmic ratio (41%)	Descriptive
	Least prevalent dysplasia features	Early keratinisation (6%) Atypical mitosis (28%) Loss of epithelial cohesion (28%)	Early keratinisation (4%) Loss of epithelial cohesion (6%) Atypical mitosis (14%)	Descriptive
DNA content (cell cycle phase)	G1	61%	68.34%	Descriptive
	S‐phase	18%	14.40%	
	G2	11%	8.1%	
	G2ER	7.3%	4.8%	
Risk stratification (MLP model)	Stratified + Fill	Low‐risk: 5 High‐risk: 13	Low‐risk: 71 High‐risk: 3	OR: 67.6 SS: 0.72 SP: 0.96 PPV: 0.81 NPV: 0.94 *p*‐value: 0.0001

*Note*: NK (not known), G1 (mean percentage of nuclei in the diploid peak), S‐phase (mean percentage of nuclei in the s‐phase area), G2 (mean percentage of nuclei in the tetraploid area), and G2ER (mean percentage of nuclei with a DNA content beyond the tetraploid area). OR (odds ratio), SS (sensitivity), SP (specificity), PPV (positive predictive value), NPV (negative predictive value). Bold text: qualitative differences between the groups. *p*‐value (from the log‐rank test applied to the Kaplan–Meier curve data).

### 3.2 Unadjusted Associations Between Individual Candidate Predictors and the Outcome (MT)

From the 27 attributes investigated herein, only 6 showed a significant correlation with MT individually, namely 4cER (*p* = 0.005, *rho* = 0.63), G2 (*p* = 0.04, *rho* = 0.39) [19], binary dysplasia grading (*p* = 0.003, *rho* = 0.30), loss of epithelial cohesion (*p* = 0.006, *rho* = 0.27), presence of an inflammatory infiltrate (*p* = 0.01, *rho* = 0.26), increased size and number of nucleoli (*p* = 0.02, *rho* = 0.24).

**FIGURE 3:**
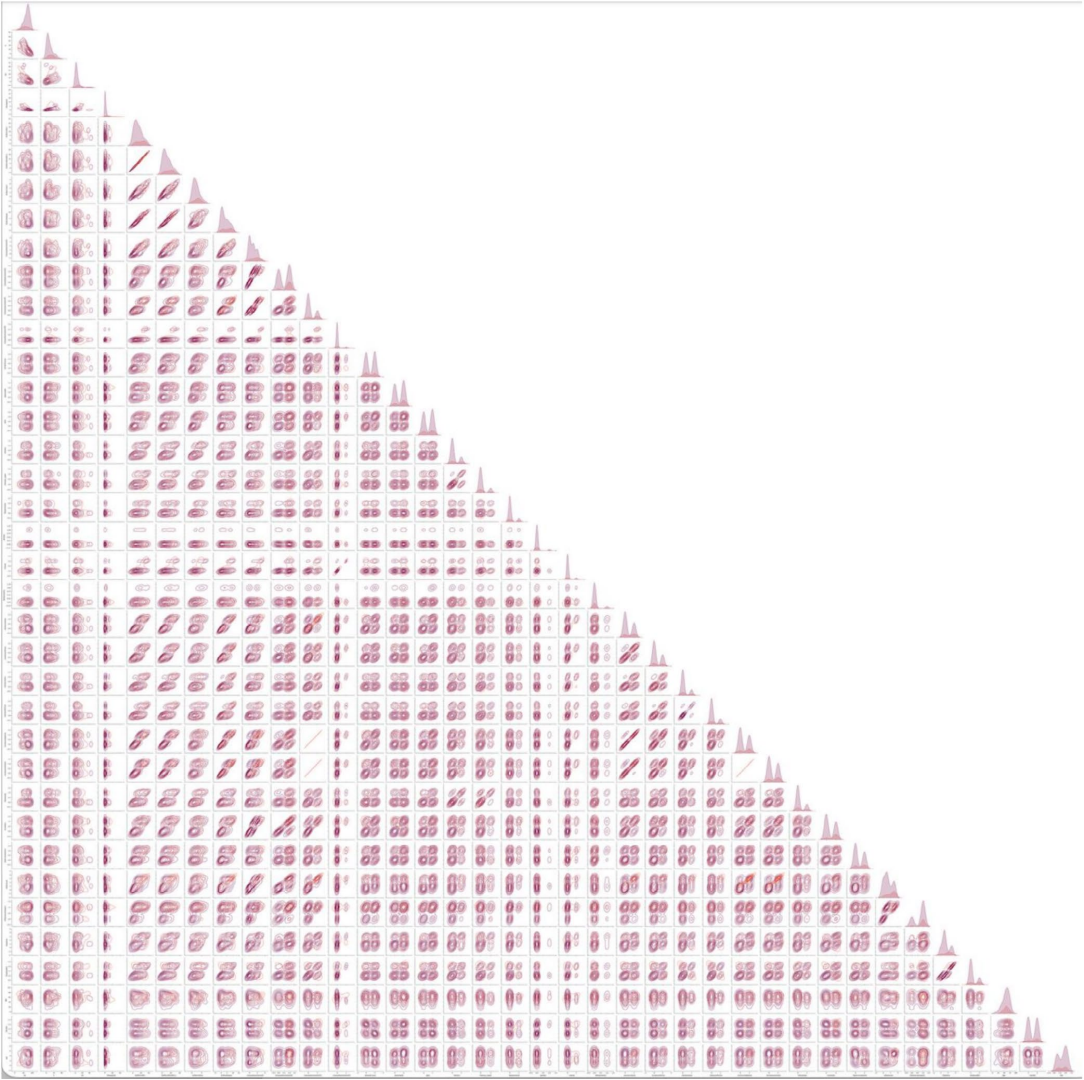
Kernel Density Plot of all attributes and combination of attributes to visually inspect the distribution of cases in the two populations: Non‐transforming (light pink) and transforming (dark pink). Note the attributes with diverging tendencies in the histograms. The attributes increased number and size of nucleoli (the transforming population increased in number in second peak (positive cases) for this variable, while the non‐transforming population decreased in number in the second peak, displaying an inverted tendency between the groups, therefore a potential feature to separate high‐risk from low‐risk cases). A similar pattern is observed for the presence of an inflammatory infiltrate, and dysplasia grading. High resolution image in Supporting Information.

The visual evaluation of the KDE distributions for each of the 27 independent variables revealed potential to differentiate the transformed subgroup from the non‐transformed subgroup for the following attributes: increased number and size of nucleoli (among the high‐risk group there were more positive cases for this variable, while in the low‐risk group fewer positive cases were present). A similar pattern was observed for the presence of an inflammatory infiltrate, and dysplasia grading (Figure 3).

### 3.3 MLP Output From the Strategies Used

**FIGURE 4:**
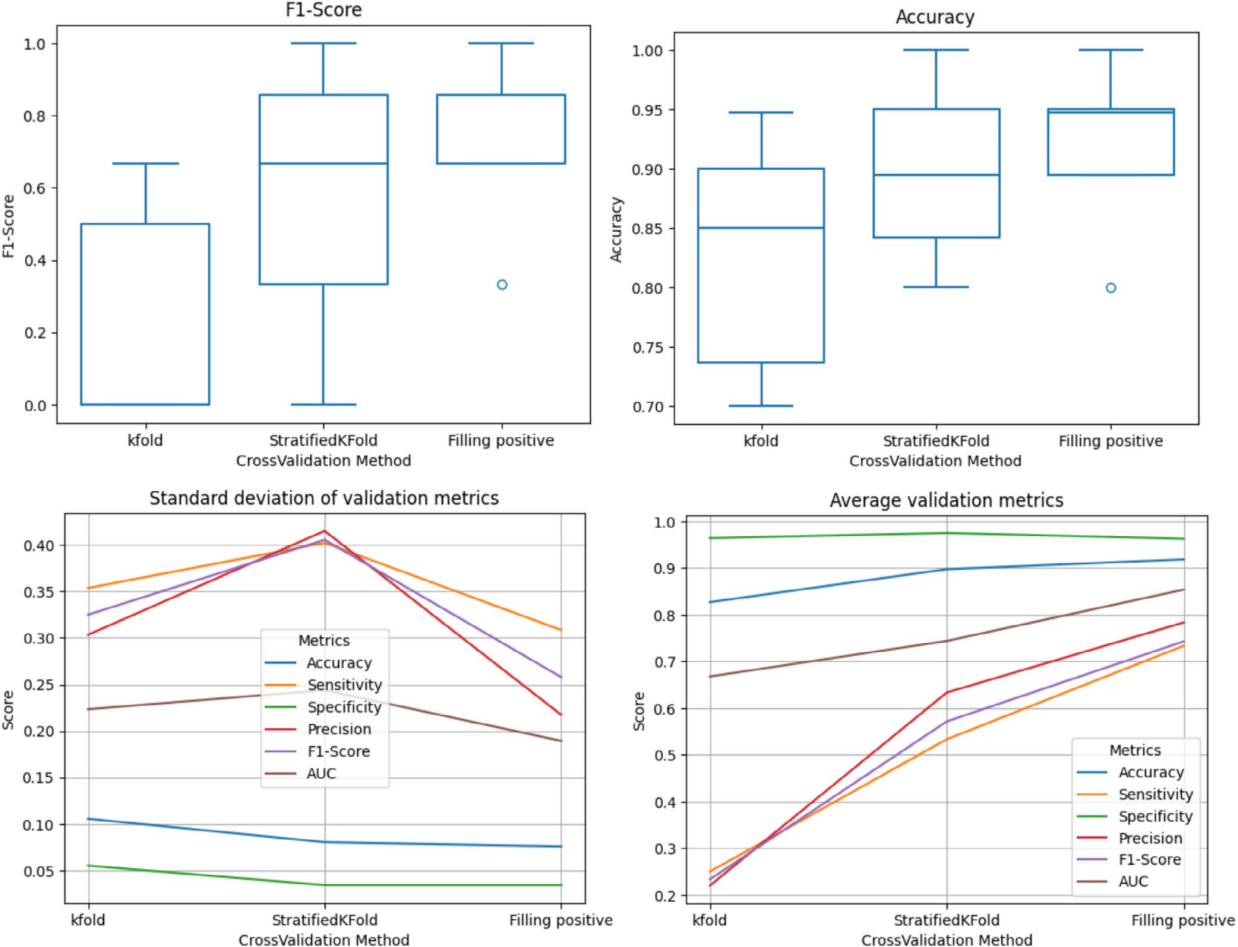
The box plot of F1‐Score and Accuracy; as well as the line plot of the three training strategies, as it can be seen the proposed Positive Filling strategy not only reached the highest values but also demonstrated the best stability by the narrow outcome distribution and low standard deviation.

The performance of the proposed MLP model across different training strategies with corresponding evaluation is summarized in Table 2. The Simple k‐fold cross‐validation achieved an average accuracy of 82.68% and exhibited significant variability in its ability to detect positive cases (MT), with a sensitivity of 25% and a large standard deviation (±35.36%). The model's specificity remained consistently high at 96.39%, reflecting its strong capacity to correctly identify negative cases (no MT). Precision (22% ± 30.33%) and F1‐Score (23.33% ± 32.49%) also displayed high variability, highlighting the model's difficulty in consistently classifying MTs, particularly due to the limited number of positive samples. The AUC was 66.76% ± 22.33%, indicating moderate performance (Table 2 and Figure 4).

**TABLE 2:** Model performance indicators for the three cross validation methods used.

Validation method	Accuracy	Sensitivity	Specificity	Precision	F1‐Score	AUC
kfold	82.68% **±** 10.56%	25.00% **±** 35.36%	96.39% **±** 5.52%	22.00% **±** 30.33%	23.33% **±** 32.49%	66.76% **±** 22.33%
StratifiedKFold	89.73% **±** 8.04%	53.33% **±** 40.22%	97.50% **±** 3.42%	63.33% **±** 41.50%	57.14% **±** 40.55%	74.37% **±** 24.38%
StratifiedKFold with positive filling	91.84% **±** 7.59%	73.33% **±** 30.84%	96.25% **±** 3.42%	78.33% **±** 21.73%	74.28% **±** 25.78%	85.36% **±** 18.93%

*Note*: Note incremental improvement in the mean performance indicators as well as reduction in standard deviation, indicating stabilization of performance variability with the stratified KFold approach with positive filling.

**FIGURE 5:**
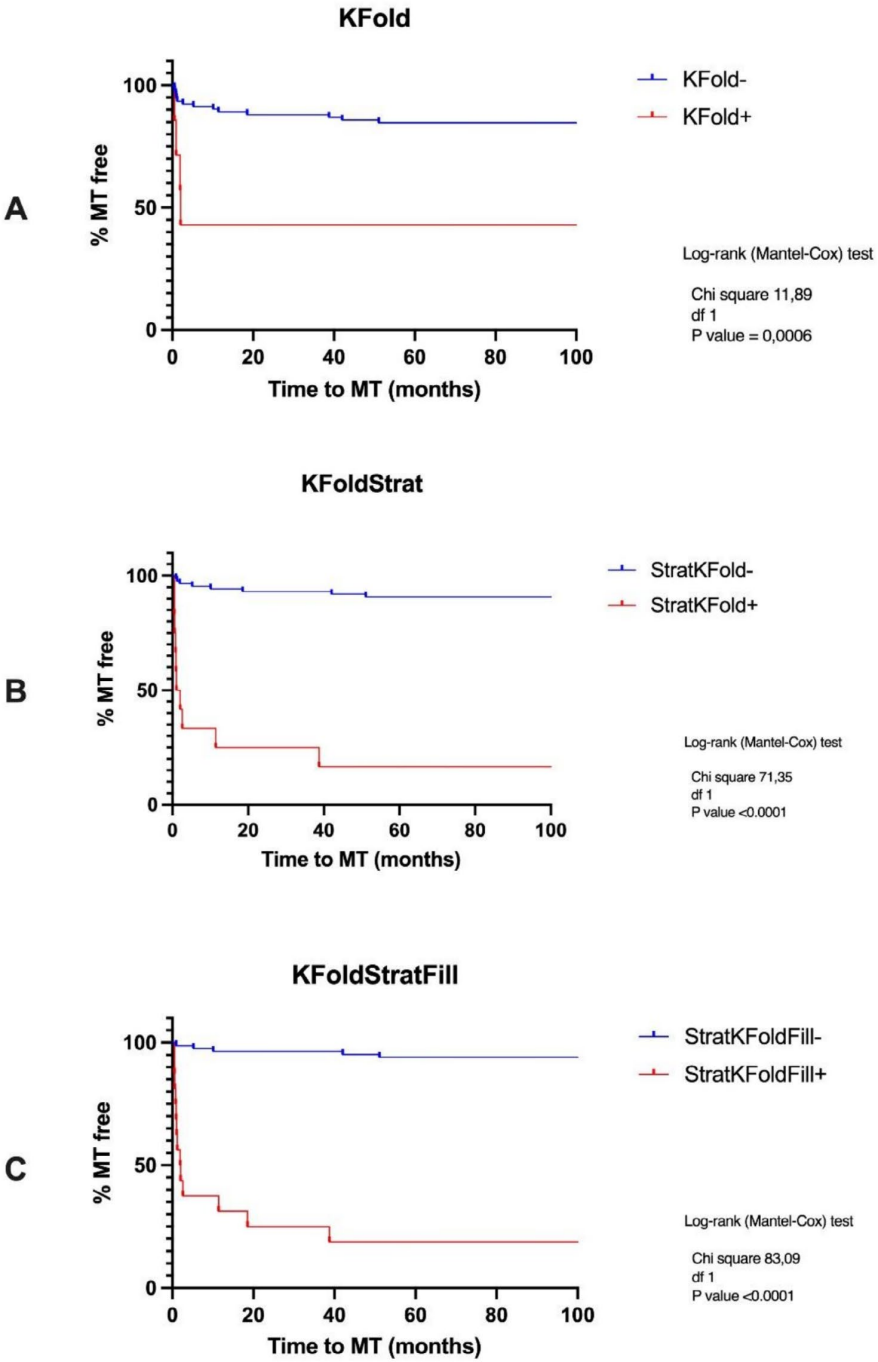
Kaplan–Meier curves of the predictive outcomes for the three cross‐validation methods used: (A) KFold, (B) Stratified KFold and (C) Stratified KFold with positive filling.

Based on the final static predictive output (not on the dynamic performance of the model described in the previous paragraph), the log‐rank (Mantel‐Cox) test detected a significant difference between the high‐risk and low‐risk distributions on the Kaplan–Meier survival curves (chi square 11.89, df 1, *p* = 0.0006), as illustrated in Figure 5A. This translated into a PPV for high‐risk and an NPV for low‐risk lesions of 57.14% and 84.78%, respectively. This also reflected in 22.2% sensitivity and 93.3% specificity.

In contrast, the Stratified KFold approach led to notable improvements in sensitivity, increasing it to 53.33% ± 40.22%, while maintaining high specificity (97.50% ± 3.42%). Precision and F1‐Score also recorded marked increases, at 63.33% ± 41.50% and 57.14% ± 40.55%, respectively. These improvements reflect the advantage of stratifying the data to maintain the proportion of malignant cases in each fold, mitigating the model's bias toward the dominant class. The AUC also increased to 74.37% ± 24.38%, showing improved discriminatory power (Table 2 and Figure 4).

Based on the final static predictive output (not on the dynamic performance of the model described in the previous paragraph), the log‐rank (Mantel‐Cox) test detected a significant difference between the high‐risk and low‐risk distributions on the Kaplan–Meier survival curves obtained from the Stratified KFold cross validation approach (chi square 71.35, df 1, *p* < 0.0001), as illustrated in Figure 5B. This translated into a PPV for high‐risk and an NPV for low‐risk lesions of 83.33% and 90.80%, respectively (Table 1). This also reflected in 55.55% sensitivity and 97.53% specificity.

The best performance was observed with StratifiedKFold with positive sample filling, where sensitivity rose to 73.33% ± 30.84%, further narrowing the gap in the detection of malignant cases. Specificity remained high at 96.25% ± 3.42%, and precision improved to 78.33% ± 21.73%, demonstrating the model's enhanced ability to correctly identify positive cases. The F1‐Score increased to 74.28% ± 25.78%, representing a more balanced performance between precision and recall. The AUC reached 85.36% ± 18.93%, reflecting significant gains in the model's overall predictive power (Table 2 and Figure 4).

Based on the final static predictive output (not on the dynamic performance of the model described in the previous paragraph), the log‐rank (Mantel‐Cox) test detected a significant difference between the high‐risk and low‐risk distributions on the Kaplan–Meier survival curves obtained from the cross‐validation approach described as Stratified KFold with positive filling (chi square 83.09, df 1, *p* < 0.0001), as illustrated in Figure 5C. This translated into a PPV for high‐risk and an NPV for low‐risk lesions of 81% and 94%, respectively (Table 1). This also reflected in 72% sensitivity and 96% specificity.

## 4 Discussion

Leveraging machine learning approaches, particularly through the integration of DNA content analysis and detailed histopathological data of individual dysplastic features, offers a promising avenue to improve the predictive accuracy of MT of OL. This study harnesses the MLP model to amalgamate multiple data layers—clinicopathological features, and DNA content—forming a robust framework for assessing the malignant potential of OL. By refining risk stratification, this model not only advances our understanding of OL progression but also holds the potential to become an indispensable tool in clinical decision‐making, aligning with the current trends toward precision medicine in cancer prognosis.

Although individual attributes were found to correlate significantly with MT, such as 4cER and G2 from DNA content (ploidy analysis) as well as some individual dysplasia features, most of them reached a weak correlation, except for 4cER, which reached a moderate correlation strength [19]. This reiterates the fact that no single marker might ever be robust enough to establish the risk of MT of OL. The more data, however, the more complex it becomes to explain how to fit a line or a curve between the independent variables and the outcomes (classes or dependent variables). It is becoming increasingly achievable to use sophisticated statistical approaches that can combine all the data and see unconventional patterns across the independent variables to classify cases into high‐risk and low‐risk of MT. The MLP used in this study combined 27 attributes to decipher a classification pattern that translated into an accurate risk assessment tool that can predict MT, especially in the cases of OL for which cancer was imminent, as depicted from the Kaplan–Meier curves. Some recent studies corroborate the importance of combining attributes [5, 7, 11, 20].

The advantages of developing combined data strategies are evident from studies that have used single (or few) markers and traditional data analysis, reporting PPV of 40% at best and those that have pooled data together and used AI‐based statistics, which demonstrated a significant increase in accuracy metrics [5, 6, 7, 20].

The high standard deviations observed in sensitivity, precision, F1‐Score, and AUC metrics highlight the difficulty of achieving consistent classification performance, especially for positive data (MT). The proposed positive filling method not only achieved the highest values but also exhibited the greatest stability, as evidenced by the narrow distribution of outcomes and low standard deviation. This result was expected, especially given the small sample size. However, the study shows that balancing the positive samples, even without adding new information to the dataset, and while maintaining the integrity of the training and test sets, significantly improved the model's training focus across classes. This adjustment resulted in more consistent outcomes across cross‐validation iterations, as evidenced by improved mean performance and lower standard deviations. Oversampling strategies are beneficial in biomedical applications with imbalanced and complex data, particularly when the minority class is clinically significant and heterogeneous enough to ascertain representativeness [5, 7, 11, 17, 18]. In our study, this strategy led to a +48% increase in sensitivity and improved performance stability, with no loss in specificity. The model had a high specificity across all methods, consistently exceeding 96%, indicating that it was effective at detecting negative cases (no MT). This balance between increasing sensitivity and maintaining specificity demonstrates the robustness of our training approach for the proposed application. For future work, we aim to further enhance performance by focusing on several key areas: (1) expanding collaborations to gain access to more original data, particularly for the underrepresented class; (2) investigating and applying advanced synthetic data generation techniques to better balance the dataset; and (3) exploring alternative models and training strategies to achieve even greater improvements in sensitivity without compromising other metrics. Such strategies are particularly important in clinical contexts where accurate detection of MT is critical. Improving sensitivity is essential in the clinical context because this metric highlights the proportion of malignant cases correctly identified by the model among all positive instances (MT). For clinical diagnosis and prognostication, it is crucial that a model does not misclassify MT cases. Therefore, a model with high sensitivity is preferable to one with high specificity, without diminishing the importance of specificity, which plays a vital role in minimizing false positives and ensuring accurate patient care.

The findings presented herein explored the potential impact of adding DNA content features to traditional histologic markers, because whole‐slide image‐based AI strategies are largely elusive in the world where oral cancer is mostly out of control, and access to machine‐led high‐end technology may take a while to materialize. In such scenarios, less objective approaches such as dysplasia grading and DNA content analysis combined could play a pivotal role in assessing risk [6, 11, 21, 22]. From a clinical standpoint, the proposed MLP model adds value beyond conventional dysplasia grading or binary classification by offering a multifactorial, data‐driven risk stratification tool that enhances early identification of high‐risk OL cases. Traditional histological grading is limited by interobserver variability and lacks sensitivity in detecting lesions with imminent MT. In contrast, our model integrates DNA content parameters (e.g., 4cER, G2 phase) and histopathological features into a cohesive predictive framework with improved accuracy and sensitivity (72%) while maintaining high specificity (96%). This enables clinicians to more confidently distinguish cases that require intensified surveillance or early intervention from those that may be managed conservatively. Importantly, the model's Kaplan–Meier stratification highlights its prognostic utility, suggesting potential application in routine follow‐up planning. As flow cytometry is increasingly accessible in tertiary settings, the model could be feasibly adopted to guide personalized patient management in real‐world scenarios, especially where high‐end digital pathology infrastructure is lacking.

Not all cases from archived tissue can be DNA‐content analyzed. Flow cytometry demands a large amount of tissue (50‐μm sections) from paraffin blocks to obtain meaningful results, which presents difficulties when working with small biopsy samples. Paraffin blocks with limited tissue often lack the sufficient material for thorough analysis, making it challenging to retrospectively assess DNA content in all clinically diagnosed OL cases using flow cytometry. A further limitation relates to the assumption of the first biopsy as the reference sample in patients with multiple lesions or serial biopsies. Recognizing the potential for spatial and temporal heterogeneity in OPMDs [23], we adopted a “first worst” approach—selecting the earliest biopsy with the highest DNA content abnormality, as described in previous studies [6, 8, 10]. While this strategy reduces the risk of underestimating malignant potential, it does not eliminate the inherent sampling bias of single‐site histological assessment. Future studies incorporating serial biopsies, whole‐slide imaging, or non‐invasive sampling may provide a more comprehensive view of lesion dynamics and biological variability.

The issue of generalizability due to representation bias is a possible challenge in this study, as the dataset only represents a specific geographic and clinical population. This can result in overfitting, which may affect the model's performance when applied to larger, more diverse populations. To address this, future research will focus on increasing data collection to include more diverse populations by performing collaborative multi‐centre studies, as well as investigating techniques such as domain adaptation and advanced synthetic data generation to improve the model's robustness. The MLP model presented herein is a proof‐of‐concept with the potential to become an additional tool in the armamentarium of risk assessment of OPMD. All ML models should therefore benefit from validation on different populations and an increased number of cases to reduce the risk of classification errors.

## 5 Conclusion

This study demonstrates the potential clinical utility of integrating nuclear DNA content analysis with machine learning to enhance risk assessment in OL. By combining objective biomarkers such as the 4c exceeding rate with key histopathological features, the multilayer perceptron model achieved high specificity and meaningful stratification of transformation risk. This approach offers a promising complement to traditional dysplasia grading, reducing subjectivity and improving predictive accuracy. In clinical practice, such a model could support more personalized surveillance strategies, aid in early intervention decisions, and optimize resource allocation by identifying high‐risk patients who warrant surgery and closer follow‐up. Future prospective validation in larger, multi‐center cohorts will be essential to confirm its generalizability and integration into routine care.

## Author Contributions

**Guilherme Iani Pontes:** methodology, software, validation, formal analysis, writing – original draft. **Anna Luíza Damaceno Araújo:** methodology, formal analysis, writing, review and editing. **Andresa Borges Soares:** conceptualization, resources, investigation, validation, writing – original draft. **Saman Warnakulasuriya:** conceptualization, writing – review and editing. **André Luis Santana de Freitas:** investigation, visualization. **Caroline Gennari Stevão:** investigation, visualization. **Marcelo Sperandio:** conceptualization, methodology, data analysis, resource and funding acquisition, data curation, supervision, project administration, ethics and compliance. **Matheus Cardoso Moraes:** conceptualization, methodology, data analysis, funding acquisition, supervision, validation, software.

## Conflicts of Interest

The authors declare no conflicts of interest.

## Data Availability

The data generated and analyzed during the current study is not publicly available due to ethical restrictions but is available from the corresponding author on reasonable request.
